# Opportunities and challenges for direct C–H functionalization of piperazines

**DOI:** 10.3762/bjoc.12.70

**Published:** 2016-04-13

**Authors:** Zhishi Ye, Kristen E Gettys, Mingji Dai

**Affiliations:** 1 Department of Chemistry and Center for Cancer Research, Purdue University, West Lafayette, Indiana 47907, United States

**Keywords:** α-lithiation, C–H functionalization, heterocycle, photoredox catalysis, piperazine

## Abstract

Piperazine ranks within the top three most utilized N-heterocyclic moieties in FDA-approved small-molecule pharmaceuticals. Herein we summarize the current synthetic methods available to perform C–H functionalization on piperazines in order to lend structural diversity to this privileged drug scaffold. Multiple approaches such as those involving α-lithiation trapping, transition-metal-catalyzed α-C–H functionalizations, and photoredox catalysis are discussed. We also highlight the difficulties experienced when successful methods for α-C–H functionalization of acyclic amines and saturated mono-nitrogen heterocyclic compounds (such as piperidines and pyrrolidines) were applied to piperazine substrates.

## Introduction

**Figure 1:**
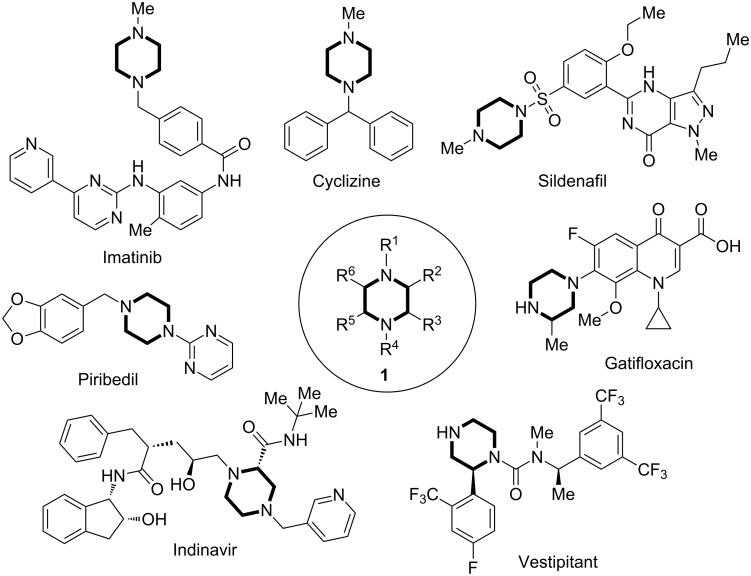
Selected piperazine-containing small-molecule pharmaceuticals.

Piperazine is one of the most important saturated N-heterocycles frequently found in life-saving small-molecule pharmaceuticals [1]. In a recent statistical study done by Njardarson and co-workers, piperazine ranks among the top three N-heterocycles along with pyridine and piperidine in the U.S. FDA-approved pharmaceuticals [2]. Due to its broad utilization, piperazine has been considered as a privileged scaffold in drug discovery to combat various human diseases (Figure 1). For example, Imatinib (also marketed as Gleevec), a BCR-Abl tyrosine kinase inhibitor, is used in the treatment of multiple cancers with high response rate [3]. Sildenafil, sold as Viagra, is an important medication for treating erectile dysfunction as well as pulmonary arterial hypertension [4]. Indinavir, a protease inhibitor, is used to treat HIV/AIDS [5]. Gatifloxacin is an important fluoroquinolone antibiotic [6]. Despite the high frequency appearance of piperazines in small-molecule pharmaceuticals, over 80% only contain substituents at the two nitrogen atoms and a very small fraction of them have simple carbon substitutions (methyl or carboxylate). Recently, other substituents such as aryl and alkyl groups started to appear on the α-carbons of piperazine rings of various important lead compounds in the pipeline of drug discovery [7–9]. Vestipitant, a neurokinin-1 antagonist, is an example which is currently in clinical trials for the treatment of anxiety and tinnitus [7]. However, such cases are rare and there is still a significant lack of structural diversity in piperazine-containing pharmaceuticals and small-molecule collections mainly due to the lack of efficient and reliable methods to quickly access carbon-substituted piperazines in regioselective and stereoselective manners.

**Figure 2:**
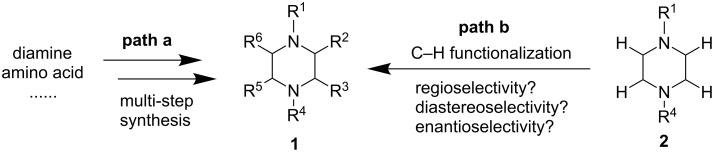
Strategies for the synthesis of carbon-substituted piperazines.

The common and traditional way to synthesize α-carbon-substituted piperazines is through de novo construction of the six-membered ring with starting materials such as amino acids and diamines followed by oxidation level adjustment (Figure 2, path a) [10]. This approach is generally lengthy, not flexible, and the substitution pattern highly depends on the availability of the starting materials. Recently, advances have been made to address some of these issues encountered in the synthesis of carbon-substituted piperazines [11]. For example, Bode and co-workers have developed a tin (Sn) amine protocol (SnAP) to synthesize piperazines and other N-heterocyles from aldehydes [12–14]. Aggarwal and co-workers have developed a formal [4 + 2] procotocl utilizing vinyl sulfonium salts and diamines as starting materials [15–17]. Carreira et al. have developed a ring expansion of 3-oxetanone to synthesize substituted piperazines [18]. Transition metal (such as Ti, Au, and Pd) catalyzed cyclizations of linear starting materials have been used by several groups including the Schafer, Nelson, Huang, and Wolfe groups to synthesize carbon-substituted piperazines [19–22]. Mendoza et al. have developed a [3 + 3] dimerization of azomethine to synthesize highly substituted piperazines [23]. Notably, Stoltz and co-workers recently developed an enantioselective synthesis of piperazin-2-ones and piperazines using a palladium-catalyzed asymmetric allylic alkylation [24]. The most straightforward and attractive way of synthesizing α-carbon-substituted piperazines is the selective (regioselective, diastereoselective, and enantioselective) activation and functionalization of the existing C–H bonds of piperazine substrates (Figure 2, path b). Although there have been major advancements made in the field of direct sp^3^ C–H bond activation and functionalization adjacent to nitrogen in saturated N-heterocycles and acyclic amines [25–27], C–H functionalization of piperazines has been a daunting challenge. In comparison to the well-studied pyrrolidine and piperidine systems, the existence of the second ring-bound nitrogen in piperazines either causes various side reactions or inhibits or diminishes the reactivity of the C–H bond. This review summarizes the current status and challenges of direct C–H bond functionalization of piperazines.

## Review

### Direct α-C–H lithiation trapping

Since the seminal discovery made by Beak and Lee [28–29], α-functionalization of *N*-Boc-protected nitrogen heterocycles via direct α-C–H lithiation trapping has been a straightforward and effective method to introduce various substituents on the α-carbon atoms [30–31]. Corresponding enantioselective versions have also been developed using chiral diamines as ligands to allow access to enantioenriched α-substituted nitrogen heterocycles. However, most of the success has been made in the territory of *N*-Boc-pyrrolidine [32–33] and *N*-Boc-piperidine [34–36], especially with regard to the asymmetric versions. The addition of the second nitrogen atom in piperazines significantly increases the reaction difficulty and complexity and only limited examples of direct α-C–H lithiation trapping of piperazines have been reported.

**Figure 3:**
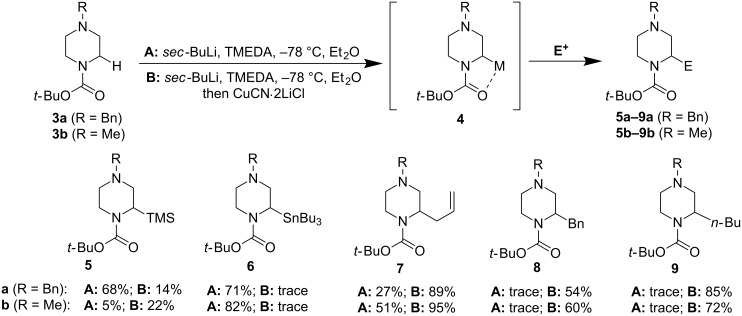
The first α-lithiation of *N*-Boc-protected piperazines by van Maarseveen et al. in 2005 [37].

The first examples of direct α-lithiation of *N*-Boc-protected piperazines were reported by van Maarseveen and co-workers in 2005 [37], sixteen years after Beak and Lee’s seminal discovery. Van Maarseveen et al. have developed two sets of reaction conditions: one uses various electrophiles to directly trap the α-lithiation product derived from treating *N*-Boc-protected piperazines with *sec*-BuLi at –78 °C (Figure 3, conditions A) while the other converts the α-lithiation product to an α-Cu intermediate via transmetallation followed by electrophilic trapping (conditions B). As shown in Figure 3, conditions A generally work better for electrophiles such as TMSCl and Bu_3_SnCl whereas conditions B are more suitable for alkyl electrophiles. Van Maarseveen and co-workers also noted that the substituents on the distal nitrogen, while lacking proximity to the reaction center, have a significant impact on the overall result.

**Figure 4:**
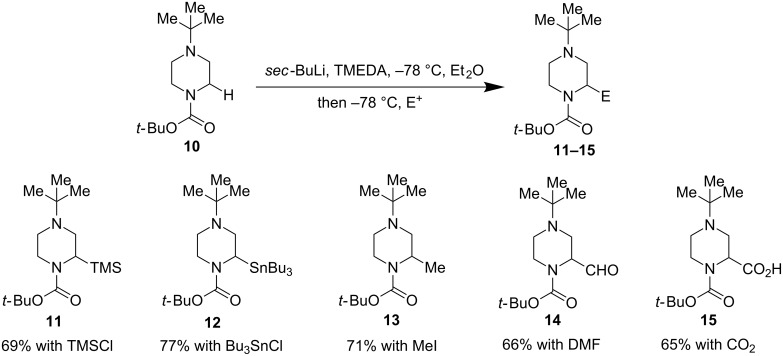
α-Lithiation of *N*-Boc-*N’*-*tert*-butyl piperazines by Coldham et al. in 2010 [38].

In 2010, Coldham and co-workers reported a direct α-functionalziation of *N*-Boc-*N*’-*tert*-butylpiperazines (Figure 4) [38]. Under the conditions of *sec*-BuLi and TMEDA, substituents such as TMS, Bu_3_Sn, Me, CHO, and CO_2_H could be installed on the *N*-Boc α-carbon in good yields. In general, the bulky *tert*-butyl group on the distal nitrogen gave better results than small alkyl groups such as methyl or benzyl groups.

**Figure 5:**
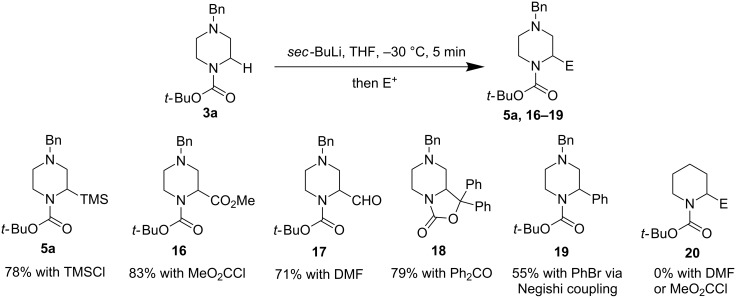
Diamine-free α-lithiation of *N*-Boc-piperazines by O’Brien, Campos, et al. in 2010 [40].

Both van Maarseveen and Coldham’s cases required the use of TMEDA and the reaction takes place at –78 °C, a reaction temperature which requires a considerable amount of energy to maintain when the reaction is conducted on a production scale (multikilogram or more) [39]. In order to circumvent these operational issues, O’Brien, Campos, and co-workers developed a diamine-free lithiation trapping process to functionalize *N*-Boc-heterocycles including piperazines using *sec*-BuLi in THF (Figure 5) [40]. This simple and effective diamine-free procedure allowed the reaction to take place at −30 °C, which is more desirable than −78 °C in process chemistry. Under the new reaction conditions, electrophiles such as TMSCl, MeO_2_CCl, DMF, Ph_2_CO, and PhBr (via a Negishi coupling process) can be used to install the corresponding substituents on the α-carbon of *N*-Boc-*N*’-benzylpiperazines in good yield. Notably, the O’Brien–Campos conditions work well for *N*-Boc-pyrrolidine and imidazolidine, but not for *N*-Boc-piperidine (cf. **20**).

### Asymmetric direct α-C–H lithiation trapping

**Figure 6:**
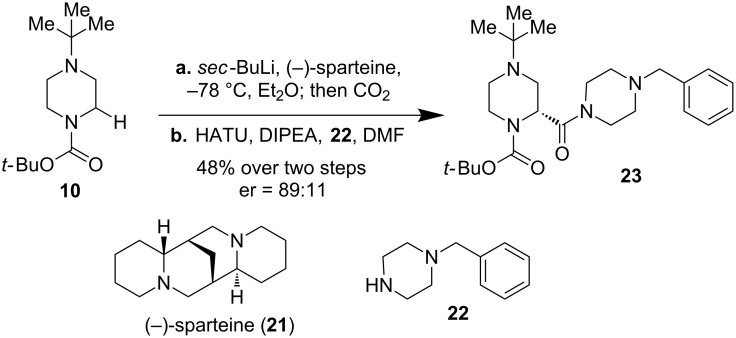
The first enantioselective α-lithiation of *N*-Boc-piperazines by McDermott et al. in 2008 [41].

Advances of enantioselective α-functionalization of *N*-Boc-protected saturated mono-nitrogen heterocycles via the lithiation trapping sequence using chiral diamines such as (−)-sparteine and (+)-sparteine surrogates as ligands have been made. However, the progress for the enantioselective α-functionalization of *N*-Boc-protected piperazines is surprisingly slow and very few examples have been reported. There remains a demand for a general versatile method to efficiently synthesize enantioenriched α-substituted piperazines. The first example of a *sec*-BuLi/(−)-sparteine-mediated asymmetric deprotonation of *N*-Boc-*N’*-*tert-*butylpiperazine was reported by McDermott et al. in 2008 (Figure 6) [41]. In two steps (asymmetric deprotonation followed by a carbon dioxide quench and coupling with *N*-benzylpiperazine, **22**) product **23** was produced in 48% yield with 89:11 enantioselectivity favoring the *R*-configuration of the newly generated carbon center.

**Figure 7:**
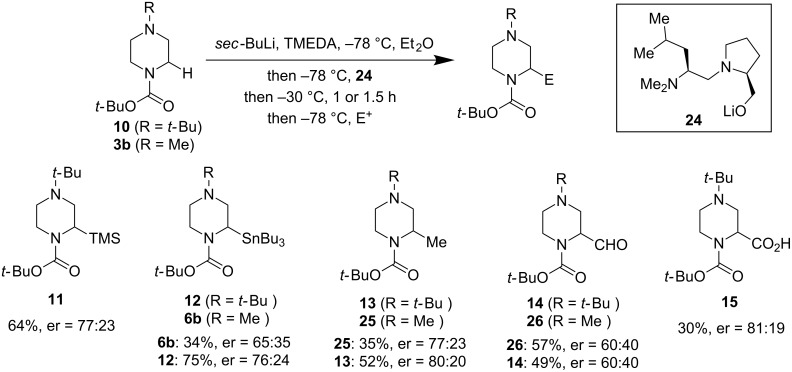
Dynamic thermodynamic resolution of lithiated of *N*-Boc-piperazines by Coldham et al. in 2010 [38].

In contrast to the direct asymmetric deprotonation, Coldham and co-workers developed a dynamic thermodynamic resolution (DTR) of the lithiated *N*-Boc-*N*’-alkylpiperazines by taking advantage of the configurational lability of the lithiated carbanion at elevated temperatures (higher than −50 °C) [38]. After generation of the lithiated *N*-Boc-*N*’-alkylpiperazines with *sec*-BuLi and TMEDA at −78 °C, the reaction was warmed up to −30 °C with addition of a chiral ligand. The chiral ligand coordinates with the racemic lithiation product to give a diastereomeric mixture which can be resolved under thermodynamic or kinetic control with electrophilic quench by a variety of electrophiles. After evaluating a few chiral diamino-alkoxide ligands, ligand **24** was identified as a superior choice. As shown in Figure 7, the result was not optimal with only 30–75% of the desired α-substituted products being obtained and the enantiomeric ratio (er) ranging from 60:40 to 81:19. The substituents are restricted to TMS, Bu_3_Sn, Me, CHO, and CO_2_H.

**Figure 8:**
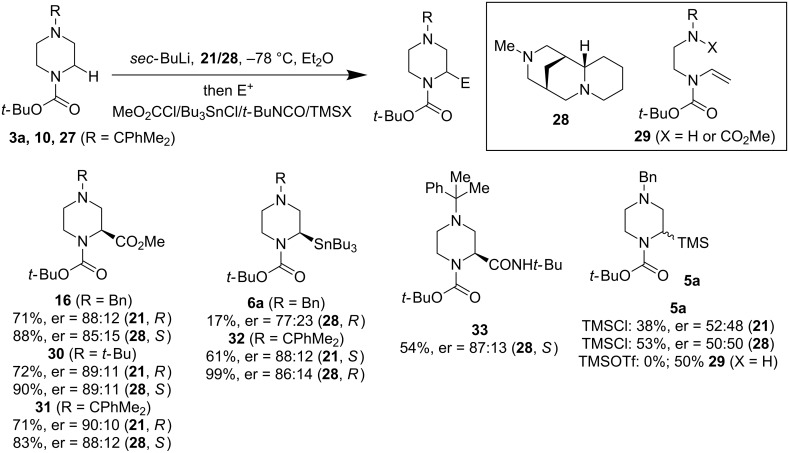
Enantioselective α-lithiation of *N*-Boc-*N*’-alkylpiperazines by O’Brien et al. in 2013 and 2016 [42–43].

O’Brien and co-workers reported another asymmetric lithiation trapping of *N*-Boc-protected saturated heterocycles at temperatures above −78 °C in 2013 [42]. Good yields and high enantioselectivity were obtained for *N*-Boc-pyrrolidines when (−)-sparteine or (+)-sparteine surrogate **28** was used. The reactions could be conducted at −30 or −20 °C with a slight drop of the enantiomeric ratio in comparison to the results at −78 °C. They also reported one example of asymmetric lithiation trapping of *N*-Boc-piperazine **10** using a combination of *sec*-BuLi and **28** to produce (*S*)-**30** with an 89:11 er. In 2015, O’Brien and co-workers expanded this work and reported an elegant and detailed study of asymmetric lithiation trapping of *N*-Boc-piperazines [43]. With the help of in situ IR spectroscopy, they were able to monitor the reaction process and establish the side reactions responsible for the observed byproduct formation (cf. **29**). As shown in Figure 8, a good to excellent selectivity could be obtained for the introduction of CO_2_Me, Bu_3_Sn, and CONH*t*-Bu groups although installation of the TMS group was found to be problematic. Similar to the Coldham discovery, they also noted that the distal *N*-alkyl substituents have a profound effect on the overall reaction yield and enantioselectivity, with the bulkier alkyl substituents giving better results. The rationale is that the bulky alkyl substituent on the distal nitrogen atom is likely preventing this nitrogen from attacking the electrophile and triggers an elimination process which would yield a byproduct like **29**.

**Figure 9:**
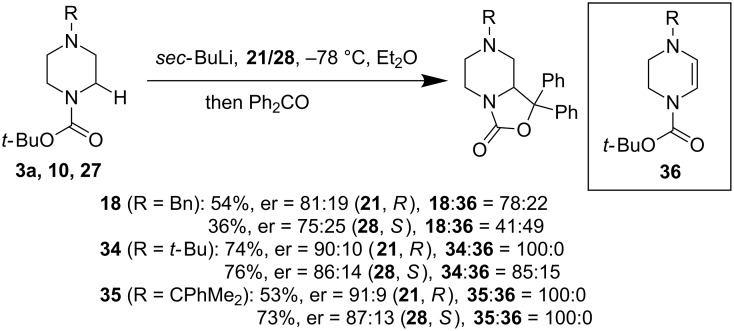
Asymmetric α-functionalization of *N*-Boc-piperazines with Ph_2_CO by O’Brien et al. in 2016 [43].

Of particular interest, when benzophenone (Ph_2_CO) was used to trap the α-lithiation product of *N*-Boc-*N*’-alkylpiperazines, in addition to the desired products (**18**, **34**, and **35**), a significant amount of oxidized product **36** was obtained (Figure 9). The formation of this byproduct is proposed to be a sequential single-electron oxidation of the alkyllithium intermediate by benzophenone. Again, the use of a bulky alkyl group on the distal nitrogen atom was seen to help reduce byproduct **36** and the desired α-functionalization product was obtained in good yield and enantioselectivity.

**Figure 10:**
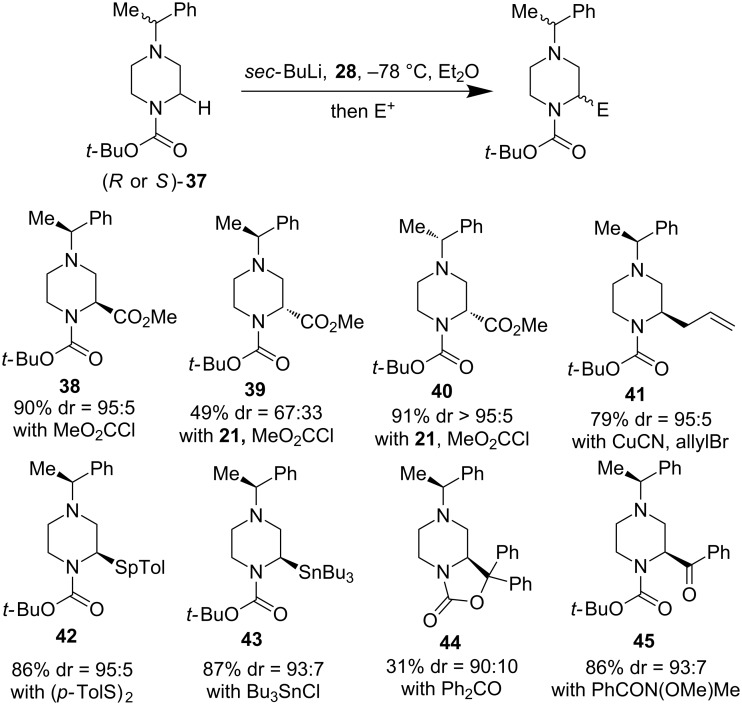
A “chiral auxiliary” strategy toward enantiopure α-functionalized piperazines by O’Brien et al. 2016 [43].

In order to prepare enantiopure α-functionalized piperazines, O’Brien and co-workers used a stereogenic α-methylbenzyl on the distal nitrogen atom. The α-methylbenzyl group is bulky enough to prevent the aforementioned side reactions and the resulting diastereomeric α-functionalized piperazines could then afford good separation. Furthermore, this “chiral auxiliary” can be removed upon catalytic hydrogenation. As shown in Figure 10, a variety of substituents could be installed on the α-position (yield of the major product was given). Notably, when (+)-sparteine surrogate **28** was used as a ligand, product **38** was produced in 90% yield with 95:5 diastereoselectivity, but once the chiral amine ligand was simply switched from **28** to (−)-sparteine (**21**), product **39** was produced in only 49% yield with poor diastereoselectivity (67:33). This result indicates a mismatched case of (−)-sparteine and the (*S*)-α-methylbenzyl group even though the chiral center of the “chiral auxiliary” is quite far away from the newly established chiral center. This issue is easily circumvented by using a matched case of (−)-sparteine and the (*R*)-α-methylbenzyl group (**40**).

**Figure 11:**
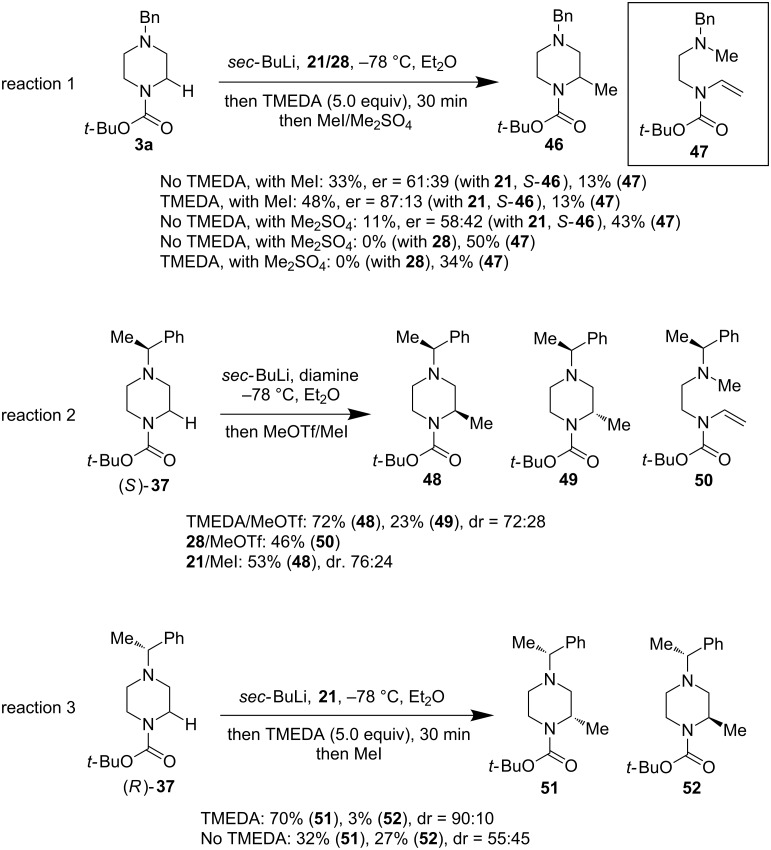
Installation of methyl group at the α-position of piperazines by O’Brien et al. 2016 [43].

The α-methyl-substituted piperazine is an important structural motif. Installation of a methyl group on a drug candidate is often seen to have a positive effect on improving the drug candidate’s activity and properties, as the so-called as the “magic methyl effect” [44]. Direct enantioselective α-methylation of piperazines however, has been a great synthetic challenge and an effective method still needs to be developed. O’Brien and co-workers also reported their work towards a solution to this problem. As shown in Figure 11, they have tried various reaction conditions to achieve an enantioselective methylation of the α-lithiation intermediate of *N*-Boc-*N*’-alkylpiperazines by using different diamines (TMEDA, **21**, and **28**) as well as the “chiral auxiliary” strategy. While the result is not yet optimal, a significant progress has been made. Due to the low reactivity of MeI and Me_2_SO_4_, a diamine switch strategy of replacing the bulky chiral diamines (**21**/**28**) with the less hindered TMEDA has been put in place to improve the reactivity of the alkyllithium intermediate and gave a 48% yield of (*S*)-**46** with an 87:13 enantiomeric ratio (Figure 11, reaction 1). The use of more reactive MeOTf with α-methylbenzyl chiral auxiliary on the distal nitrogen atom was not fruitful (Figure 11, reaction 2); a significant amount of elimination product **47** or **50** was produced in these cases. To date, a combination of the diamine switch strategy and α-methylbenzyl chiral auxiliary strategy has been reported to give the best results and produce **51** in 70% yield and 90:10 diastereoselectivity (Figure 11, reaction 3).

**Figure 12:**
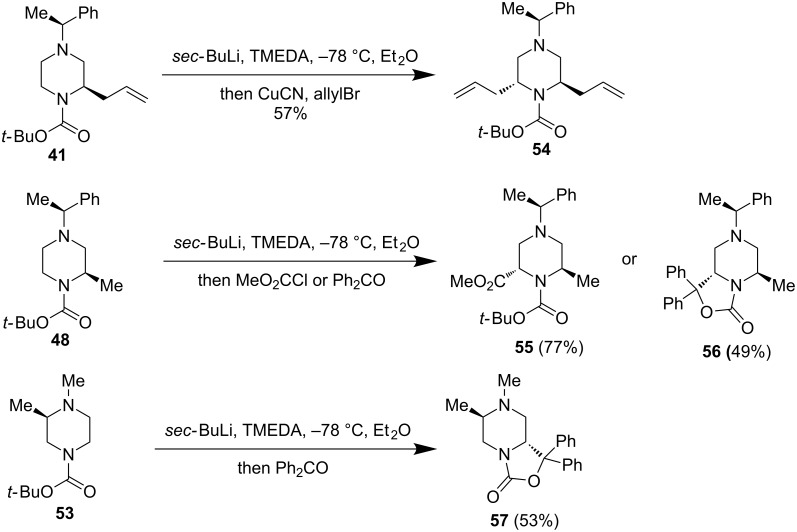
α-Lithiation trapping of *C*-substituted *N*-Boc-piperazines by O’Brien et al. 2016 [43].

O’Brien and co-workers also reported a stereoselective synthesis of enantiopure 2,6-*trans*- and 2,5-*trans*-piperazines via a second α-lithiation trapping of carbon-substituted *N*-Boc-piperazines (Figure 12). In the cases of **41** and **48**, 2,6-*trans*-piperazine products **54**, **55**, or **56** were produced in excellent stereroselectivity and good yield. The predominant formation of the 2,6-*trans*-piperazine products is presumably due to a Boc-directed equatorial lithiation trapping with the existing allyl or methyl group in the axial position to avoid strong A^1,3^-interaction with the Boc group. In the case of **53**, an equatorial lithiation trapping with the existing methyl group in the equatorial position gave 2,5-*trans*-piperazine **57**.

In summary, promising progress has been made in the direct α-lithiation trapping of *N*-Boc-protected piperazines, including enantioselective versions. So far, these methods are limited by narrow electrophile scopes and often low enantioselectivities rendering further developments necessary.

### Transition-metal-catalyzed α-C–H functionalization

Transition-metal-catalyzed direct sp^3^ C–H bond functionalization at the α-carbon of both cyclic and acyclic amines have been a fertile research field [45–47]. In the case of saturated *N*-heterocycles however, most of the efforts have been focused on directed α-C–H functionalization of pyrrolidines and piperidines [48–50]. Little progress has been made in transition-metal-catalyzed α-C–H functionalization of piperazines presumably due to the low reactivity and the undesired competitive pathways caused by the addition of the second nitrogen in the six-membered ring [51]. As of yet only a few examples have been reported so far and are far from being general and practical; no enantioselective versions have been shown.

#### Rhodium-catalyzed dehydrogenative carbonylation

**Figure 13:**
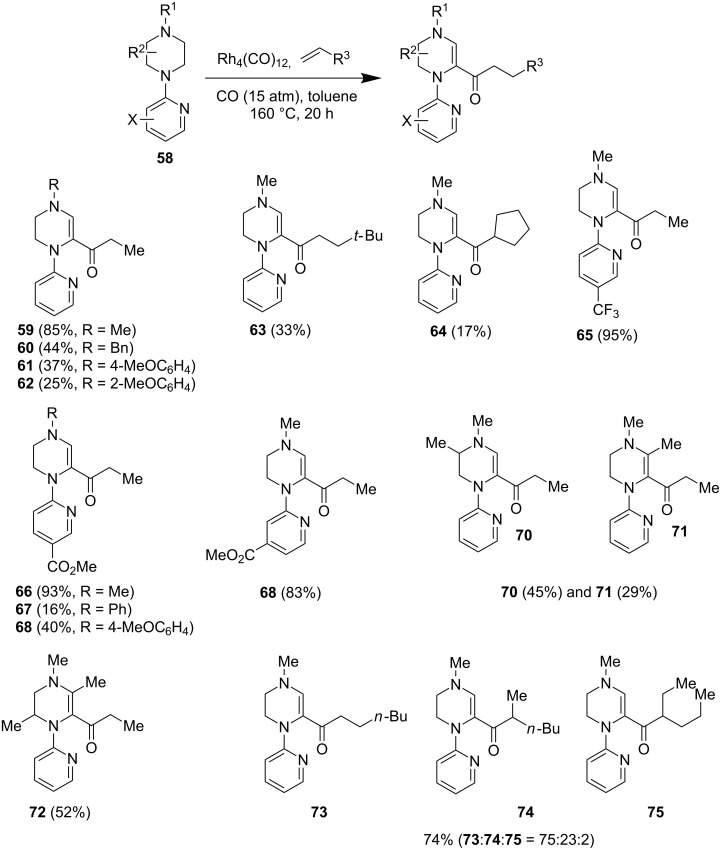
Rh-catalyzed reactions of *N*-(2-pyridinyl)piperazines by Murai et al. in 1997 [52].

In 1997, Murai and co-workers reported a novel Rh-catalyzed α-C–H-functionalization reaction of *N*-(2-pyridinyl)piperazines with carbon monoxide and terminal olefins (Figure 13) [52]. Their previous work on pyridinyl group-directed Rh-catalyzed carbonylation at sp^3^ C–H bonds adjacent to the nitrogen atom in other alkylamines such as pyrrolidine, piperidine, and tetrahydroisoquinoline [53–54] gave the carbonylation product directly. However, when piperazine substrates were used, an additional formal dehydrogenation process took place before the carbonylation reaction. As shown in Figure 13, under the conditions of 15 atm of carbon monoxide and ethylene, Rh_4_(CO)_12_ catalyst, and toluene at 160 °C, dehydrogenation and propionylation of *N*-(2-pyridinyl)piperazines took place to give various tetrahydropiperazines. Similar to the α-C–H lithiation trapping strategy, the substituents on the distal nitrogen have a profound effect on the overall yield with alkyl groups giving better yields than aryl and acyl groups. Other olefins such *tert*-butylethylene and cyclopentene can be used as well, but the yields were significantly lower (cf. **63** and **64**). The scope of the directing 2-pyridinyl group can be expanded to electron-withdrawing groups such as ester and trifluoromethyl, while maintaining excellent yields. Regioselectivity issues have been encountered in the case of C_3_-substituted piperazine substrates resulting in a mixture of **70** and **71**. Notably, when 1-hexene was used, a mixture of **73**–**75** was produced. When the 2-pyridinyl directing group was switched to simple aryl groups, only dehydrogenation products were observed. Overall, the result is promising, but this method has quite a limited substrate scope and yields only dehydrogenated products after C–H functionalization instead of the desired fully saturated piperazines. It also highlights the challenges provided by the extra nitrogen of piperazine in comparison to pyrrolidine and piperidine substrates.

#### Tantalum-catalyzed hydroaminoalkylation

**Figure 14:**
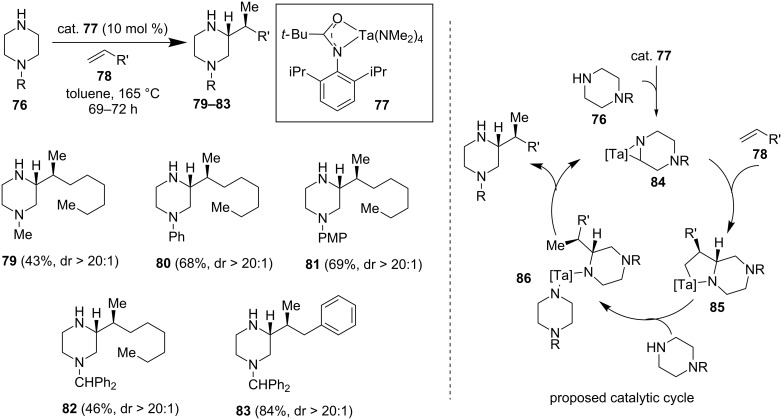
Ta-catalyzed hydroaminoalkylation of piperazines by Schafer et al. in 2013 [55].

In continuation of their efforts in developing new catalysts and methods for hydroaminoalkylation at the α-position of amines, Schafer and co-workers reported an elegant tantalum-catalyzed hydroaminoalkylation for the synthesis of α-alkylated *N*-heterocycles from the corresponding heterocycles and alkenes [55]. Along with piperidine and azepane substrates, piperazine substrates of type **76** react smoothly with terminal olefins (**78**) in the presence of 10 mol % of catalyst **77** in toluene at 165 °C (Figure 14). Despite the high temperature, the reaction provided α-alkylated piperazines **79–83** in good yield. The reaction is atom-economic and does not require directing groups which sets it apart from previously discussed models. Simple mono-alkylated or -arylated piperazines and terminal olefins were used as starting materials. The reaction proceeds with excellent regio- and diastereoselectivity which is presumably due to a regio- and stereoselective alkene insertion into the strained metalla-aziridine intermediate **84**.

#### Photoredox catalysis

**Figure 15:**
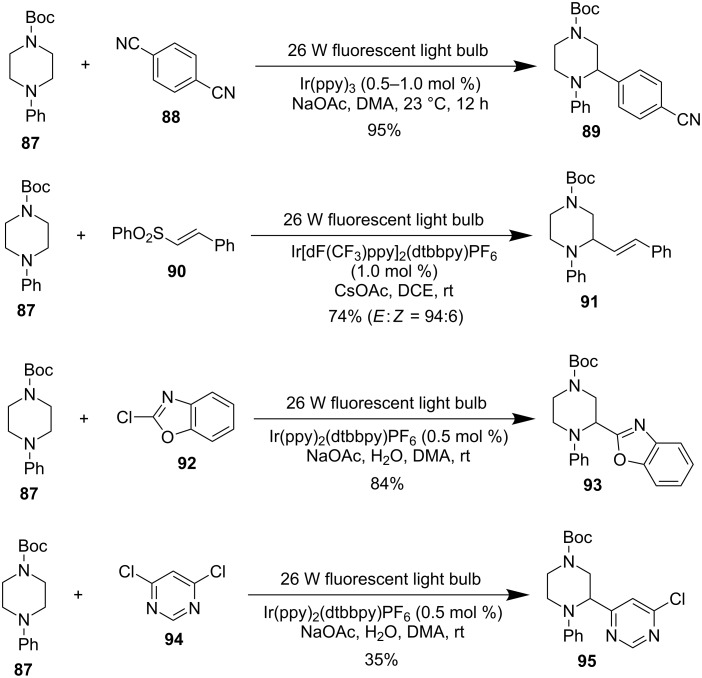
Photoredox catalysis for α-C–H functionalization of piperazines by MacMillan et al. in 2011 and 2014 [63,65–66].

Visible-light photoredox catalysis has emerged as a powerful platform for organic small-molecule functionalization [56–58]. One important application of photoredox catalysis is direct sp^3^ C–H activation and functionalization [59–61]. Among the recent advances, direct photoredox redox C–H activation of the α-position of amines has been an efficient and versatile method to functionalize amines, particularly saturated N-heterocycles [62–66]. However, photoredox catalysis for direct α-C–H functionalization of piperazines is very limited and only a few examples have been reported by MacMillan and co-workers (Figure 15) [63,65–66]. Using a high-throughput and automated workflow platform, they have discovered a photoredox-catalyzed C–H arylation of *N*-arylamines with 1,4-dicyanobenzene (**88**) to produce pharmaceutically important benzylic amines. This reaction works well with piperazine substrate **87** to synthesize the α-arylated piperazine **89** in 95% yield with Ir(ppy)_3_ as the catalyst [63]. In another report, MacMillan et al. showed that under similar photoredox conditions, **87** could couple with vinyl sulfone **90** to provide α-vinylation product **91** in 74% yield with excellent *E*/*Z* selectivity [65]. They also discovered that piperazine **87** could couple with heteroaryl chlorides **92** and **94** to obtain products **93** and **95** in 84% and 35% yield, respectively [66]. These results represent a breakthrough in the direct α-C–H functionalization of piperazines. The generation and trapping of the α-amino radical derived from **87** with radical acceptors under mild photoredox catalysis conditions could avoid the aforementioned side reactions associated with direct α-lithiation trapping and transition-metal-catalyzed C–H functionalization of piperazines. However, the involvement of an α-amino radical in the reaction process adds another layer of difficulty in achieving enantioselective versions of these transformations.

#### Copper-catalyzed C–H oxidation

**Figure 16:**
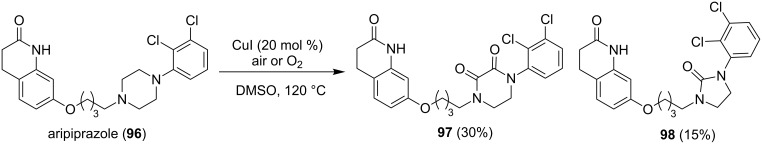
Copper-catalyzed aerobic C–H oxidation of piperazines by Touré, Sames, et al. in 2013 [67].

In an effort to establish a chemical toolkit to rapidly deliver oxidized drug metabolites, Touré, Sames and co-workers have discovered that piperazines could be oxidized to diketopiperazines as well as ring fragmented products under aerobic conditions with copper salt catalysts [67]. For example, when the antipsychotic drug aripiprazole (**96**) was treated with a catalytic amount of CuI under air or oxygen in DMSO at 120 °C, 2,3-diketopiperazine **97** was produced in 30% yield along with a 15% yield of urea product **98** (Figure 16). This method, despite its relatively low yield and selectivity, does offer a rapid way to access potential drug metabolites or analogs for further biological evaluations.

#### Free radical approach

**Figure 17:**
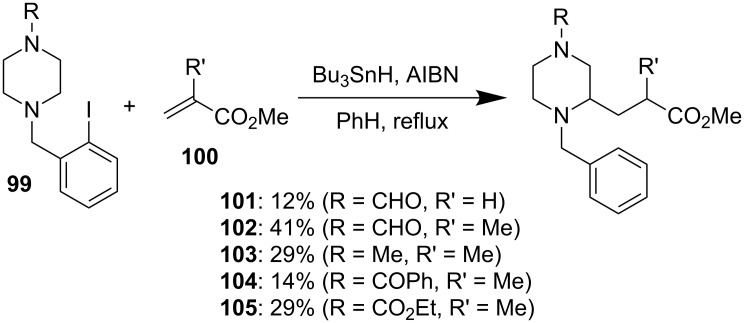
Free radical approach by Undheim et al. in 1994 [68].

In 1994, Undheim and co-workers developed a radical relay strategy, using a progression from an aryl radical to an α-amine radical followed by trapping with acrylate, to functionalize the α-position of amines [68]. The strategy works for morpholine and piperazine substrates, but the yields for the latter are generally low, ranging from 12% to 41% (Figure 17).

#### Anodic oxidation strategy

**Figure 18:**
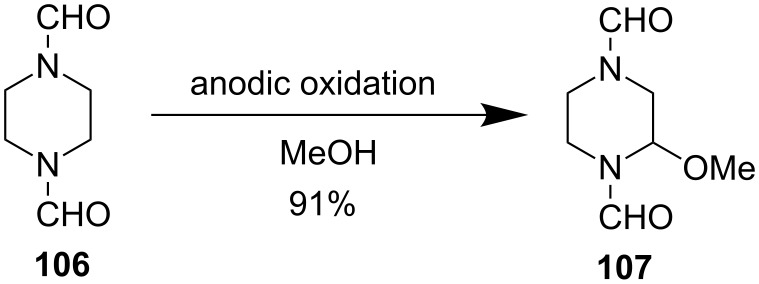
Anodic oxidation approach by Nyberg et al. in 1976 [70].

Another uncommon way to perform α-position functionalization is using electroorganic chemistry [69]. As shown in Figure 18, bisformyl protected piperazine **106** could be converted to **107** in 91% yield under anodic oxidation conditions at 500 g scale [70]. While this method is limited and only allows for functionalization with alkoxy groups, the resulting aminal products can be further diversified into other carbon-substituted piperazine products.

## Conclusion

In summary, despite the importance and necessity of substituted piperazines in medicinal chemistry, there is still a lack of general and practical methods to directly and stereoselectively introduce substituents on the α-carbons of piperazines. So far most of the efforts focus on direct α-C–H lithiation trapping, transition-metal-catalyzed α-C–H functionalization of piperazines, and photoredox catalysis. While some progress has been made, there is still a long way to go, as most of these methods are far from general and practical. Many of the known methods have a very narrow substrate scope and give poor reaction yields. The enantioselective C–H functionalization of piperazines has been a barren field despite the advances made with other saturated N-heterocycles. The addition of the second nitrogen makes piperazine behave very differently in comparison to the corresponding pyrrolidine and piperidine systems. It either creates various side reactions such as the undesired elimination or dehydrogenation pathways or diminishes the reactivity of the α-C–H bond. In order to overcome these intrinsic reactivity issues, new synthetic methods and novel catalyst systems are necessary. These engagements are important because general and practical regioselective, diastereoselective, and enantioselective C–H functionalizations of piperazines are expected to significantly enhance the structural diversity and availability of piperazine-containing small-molecule collections in the pharmaceutical industry.
